# *Ixodiphagus hookeri* (Hymenoptera: Encyrtidae) and Tick-Borne Pathogens in Ticks with Sympatric Occurrence (and Different Activities) in the Slovak Karst National Park (Slovakia), Central Europe

**DOI:** 10.3390/pathogens13050385

**Published:** 2024-05-07

**Authors:** Veronika Blažeková, Michal Stanko, Hein Sprong, Robert Kohl, Dana Zubriková, Lucia Vargová, Martin Bona, Dana Miklisová, Bronislava Víchová

**Affiliations:** 1 Laboratory of Molecular Ecology of Vectors, Institute of Parasitology, Slovak Academy of Sciences, Hlinkova 3, 040 01 Košice, Slovakia; sirotnakova@saske.sk (V.B.); stankom@saske.sk (M.S.); zubrikova@saske.sk (D.Z.); lvargova@saske.sk (L.V.); miklis@saske.sk (D.M.); 2 Department of Epizootiology, Parasitology and Protection of One Health, University of Veterinary Medicine and Pharmacy in Košice, Komenského 73, 040 81 Košice, Slovakia; 3 Institute of Zoology Slovak Academy of Sciences, Dúbravská cesta 9, 845 06 Bratislava, Slovakia; 4 Centre for Infectious Disease Control, National Institute for Public Health and the Environment, Antonie van Leeuwenhoeklaan 9, 3721 MA Bilthoven, The Netherlands; hein.sprong@rivm.nl (H.S.); robert.kohl@rivm.nl (R.K.); 5 Department of Medical Physiology, Faculty of Medicine, Pavol Jozef Šafárik University in Košice, Trieda SNP 1, 040 11 Košice, Slovakia; martinbonask@gmail.com

**Keywords:** *Ixodiphagus hookeri*, *Ixodes ricinus*, *Haemaphysalis* spp., *Dermacentor* spp., wasp, tick, Slovak Karst, pathogens, bacteria, symbionts

## Abstract

Ticks are involved in the transmission a plethora of pathogens. To effectively control ticks and mitigate the risks associated with tick-borne diseases, it is important to implement tick control measures. These may include the use of acaricides as well as the development and implementation of an alternative, environmentally friendly tick management program that include practices such as habitat modification or establishing biological control. *Ixodiphagus hookeri* Howard is a tick-specific parasitoid wasp that predates on several species of ixodid ticks and could contribute to the control of the tick population. This work aimed to detect the presence of parasitoid wasps in ticks (*Ixodidae*) using genetic approaches. Several tick species of the genera *Ixodes*, *Haemaphysalis,* and *Dermacentor*, with a sympatric occurrence in the Slovak Karst National Park in southeastern Slovakia, were screened for the presence of wasps of the genus *Ixodiphagus.* The DNA of the parasitoids was detected in four tick species from three genera. This work presents the first molecular detection of parasitoids in two *Dermacentor* tick species, as well as the first molecular identification of *Ixodiphagus* wasps in *Ixodes ricinus* and *Haemaphysalis concinna* ticks from the Karst area. In the given area, it was observed that *I. ricinus* and *H. concinna* ticks are hyper-parasitized by wasps. Moreover, it was observed that wasps here can parasitize several tick species, some of which are of less significance for human and animal health (as they transmit fewer pathogens).

## 1. Introduction

The epidemiological significance of tick genera and species can vary considerably. While some species transmit fewer microorganisms, other tick species have a broad transmission competence and significantly impair society’s economy and public health. The vector competence of individual tick species for pathogens appears to be related to its microbiome [1,2,3,4,5]. Ticks not only transmit harmful pathogens but also host several symbiotic bacteria. These interact with each other and might have beneficial, neutral, or detrimental effects on their hosts. Endosymbionts may be involved in the physiological processes of a tick host, such as survival, development, fitness, reproduction, nutritional adaptations, defense against environmental stress, and immunity [5].

For tick control, it is crucial to understand the diverse interactions between members of the microbiome. These can increase the risk of infection by promoting the transmission or multiplication of certain species of vector-borne pathogens or can lead to the elimination of pathogenic species in co-infections because of competitive exclusion [5,6,7,8,9]. The microbiota in ticks is unstable and changes over time, mainly due to differences in the compositions of the “environmental” taxa [5,10]. The composition of tick microbiota is linked to and influenced by the presence of certain species of tick-borne pathogens. Conversely, several non-pathogenic microorganisms may affect the epidemiology of vector-borne diseases [5]. However, limited information exists about these interactions in *I. ricinus* microbiota. Even less or no data are available regarding other species [11].

Currently, tick control primarily relies on acaricides, but due to resistance, toxicity, and potential environmental harm, many synthetic formulations are no longer used [12,13]. Instead, the use of botanically based substances such as plant extracts, essential oils, and biological agents as alternatives to chemical pesticides is desirable. Moreover, ticks have various natural enemies, including birds (e.g., oxpeckers of the family Buphagidae), entomopathogenic bacteria, protozoa, or fungi (such as the genera *Metarhizium* and *Beauveria*), entomopathogenic nematodes, and parasitic wasps, which could also be used as alternatives in the fight against ectoparasites [14,15,16].

Encyrtid wasps of the *Ixodiphagus* spp. (Encyrtidae, Hymenoptera) are known to parasitize a broad range of tick species (Ixodida) across the world [8,17,18]. The wasp’s life cycle is synchronized with the stage-specific activities of juvenile ticks in nature [19]. Multiple female wasps can parasitize a single tick host simultaneously after locating them probably by using the tick and/or vertebrate host’s odor cues [11,19,20,21]. In regions with a mild climate, where the tick hosts are unavailable during the winter, wasps primarily lay eggs inside the bodies of unfed tick nymphs and remain in diapause [22,23,24]. The blood’s circulation, following a blood meal of a tick on the host, induces the embryonic development of the wasps, and the diapause of the eggs is terminated [8,17]. After hatching, the wasp larvae feed on the tick’s tissue and vertebrate blood ingested by the ticks [25,26]. One to two months after oviposition, adult wasps exit the empty tick body through a single hole at the posterior end of the abdomen [8,19,27].

This study aimed to detect the presence of parasitoid wasps in ticks (Ixodidae) using genetic approaches. Several tick species of the genera *Ixodes*, *Haemaphysalis*, and *Dermacentor*, with a sympatric occurrence in the Slovak Karst National Park in southeastern Slovakia, were screened for the presence of wasps of the genus *Ixodiphagus*. Additionally, this study aimed to evaluate the range of tick-borne microorganisms that circulate in the natural foci. A comparison of the diversity of endosymbionts, pathogens, and parasites in ticks parasitized by wasps versus ticks without wasps could provide insights into the relationship between the microbes and the potential influence of the presence of parasitoids on tick-borne infections.

## 2. Material and Methods

### 2.1. Study Area

The study area is situated in the largest Karst plain in Central Europe, in the southwestern part of eastern Slovakia. It is a warm and moderately humid region with cold winters (January temperature < −3 °C). The mean number of summer days per year is >50 [28]. Diverse environmental conditions at the site determine the coexistence of several endemic species of animals and plants, which are typical of mountain, forest, or steppe landscapes. The collection site was established in the area situated on the northern grassy slope of the “Dolný vrch” hill, which is covered with scattered islands of xerophilous shrubs (212 m a.s.l., 48°34′53″ N, 20°46′44″ E), near Hrhov village (Slovak Karst National Park). It is a recreational area with a significant diversity of game animals (e.g., roe deer/*Capreolus capreolus*, red deer/*Cervus elaphus*, and wild boar/*Sus scrofa*). The location is mostly used for hunting purposes and as pastures for farm animals (mainly cattle). The sympatric occurrence of 7 tick species, namely *I. ricinus*, *I. trianguliceps*, *I. frontalis, Dermacentor reticulatus*, *D. marginatus*, *Haemaphysalis inermis*, and *H. concinna*, with an allochronic or asynchronous peak activity was recorded on the site.

### 2.2. Tick Sampling

Ticks were collected every month during the vegetation period from 2012 to 2022. The temperature and relative humidity of the environment were measured year round at the location using electronic devices (Reed R6030 with an accuracy of 1 °C and 1% RH). Flagging of the vegetation using a white cloth flag with a size of 1 × 1 m mounted on a wooden stick was implemented during tick collection [29]. The same methodological procedure was followed for each collection, which lasted for at least one hour. The tick collection was usually performed between 9:00 and 11:00 am. After every 1 to 2 m of flagging, the flag’s sides were checked for the presence of ticks. The ticks were collected, stored, and transported to the laboratory in 70% ethanol. In the laboratory, they were identified by the species and gender using a taxonomic key [30].

Tick specimens that were not used in this study were included in several previously published eco-epidemiological studies [31,32,33,34].

During these 10 years, each sampling year included approximately 30 adult ticks, 30 nymphs, and 10 pools of 10 *I. ricinus* larvae for molecular analyses. A total of 1603 *I. ricinus* ticks, which included 303 adults (154 males and 149 females), 300 nymphs, and 100 pools of 10 larvae each were analyzed.

In total, 1240 *H. concinna* (3 adult females, 297 nymphs, and 94 pools of 10 larvae each) ticks were tested.

Altogether, 124 *H. inermis* (60 females, 55 males, and 9 nymphs), 57 *D. reticulatus* (31 females, 19 males, and 7 nymphs), and 30 *D. marginatus* (12 males, 7 females, 8 nymphs, and 12 larvae) ticks collected from vegetation within the sampling period were analyzed.

Additionally, we analyzed 2 *H. inermis* larvae, which were removed from trapped *Apodemus* spp., 19 *I. trianguliceps* larvae from *Apodemus* spp., 95 larvae of *D. marginatus* from *Apodemus* spp. and *Clethrionomys glareolus*, as well as 72 *D. reticulatus* larvae from *Apodemus* spp., a *Clethrionomys* sp., and white-toothed shrew (*Crocidura leucodon*) to determine the presence of wasps and microbes. Rodent trapping methods are described in the works of Heglasová et al. [32,33] and were realized based on permission from the Ministry of Environment of the Slovak Republic, No. 4559/2015-2.3. The questing adult ticks and nymphs, as well as engorged juvenile ticks from small mammals, were processed separately, and the prevalence rates for wasps and microbes were calculated. For the pools of questing larvae, the minimum infection rate (MIR) was calculated as the ratio of the number of positive pools to the total number of larvae tested. The MIR assumes that only one infected individual exists in a positive pool [35].

### 2.3. DNA Extraction

Genomic DNA was extracted from the questing ticks using alkaline hydrolysis [36]. For the engorged ticks that were removed from the rodents captured at the model site for Heglasová et al.’s [32] study, the total DNA was extracted from each tick separately after an overnight incubation with a Proteinase K solution at 56 °C using the NucleoSpin Tissue kit (Macherey-Nagel, Düren, Germany), according to the manufacturer’s instructions.

All DNA samples were stored at −20 °C until further processing. The concentration of genomic DNA isolated from ticks was measured in a group of randomly chosen samples using a NanoPhotometer™ UV/Vis Spectrophotometer (Implen GmBH, Munich, Germany).

### 2.4. PCR Screening

Questing *I. ricinus* and *H. concinna* ticks and engorged ticks (*Dermacentor* spp., *H. inermis*, and *I. trianguliceps*) from rodents were examined using conventional PCR assays with genus-specific or species-specific primer pairs, according to published protocols (Table 1). Initially, all genomic DNA samples were screened for parasitoid wasps, *Ixodiphagus* (*Iph.*) *hookeri*, by amplifying fragments of the mitochondrial gene for cytochrome oxidase subunit 1 (*cox1*) and the ribosomal 28S rRNA gene. Subsequently, the DNA samples were examined for the presence of endosymbiotic bacteria of the *Wolbachia* spp., as well as pathogens or endosymbionts from the following genera: *Borreliella*, *Anaplasma*, *Bartonella*, *Babesia* and *Rickettsia*.

Questing *Dermacentor* spp. and *H. inermis* ticks from vegetation were examined for the presence of *Iph. hookeri* using a qPCR assay targeting the 104 bp fragment of *cox1*, according to Krawczyk et al. [37]. The presence of *Borreliella* spp./*Borrelia* spp. was detected using DNA duplex qPCR, which targets the flagellin gene, and the *B. burgdorferi* sensu lato-specific *OspA* gene encoding the outer surface protein A, according to de Leeuw et al. [38]. Subsequently, all qPCR-positive samples from *OspA*/*flaB* duplex qPCR were examined using conventional PCR to amplify the intergenic spacer region (IGS) of *B. burgdorferi* s.l., according to Coipan et al. [39], and sequenced. Three multiplex qPCR assays were used for the detection of *Anaplasma* spp., *Babesia* spp., *Bartonella*, and *Rickettsia* spp., as published by Azagi et al. [40].

**Table 1:** PCR primers and conditions used for molecular detection of pathogens in questing *I. ricinus* and *H. concinna* ticks and *Dermacentor* spp., *H. inermis*, and *I. trianguliceps* ticks removed from rodents, using conventional PCR assays.

Pathogen	Target Gene	Length (bp)	Primer Name	Primer Sequence (5′–3′)	PCR Annealing Temperature	Reference
Bacteria						
*Anaplasma* *phagocytophilum*	*msp2*	334	MSP2f	CCA GCG TTT AGC AAG ATA AGA G	55 °C	[41]
			MSP2r	GCC CAG TAA CAT CAT AAG C		
*Borreliella* spp.	*rrfA-rrlB*	222–255	IGSa	CGA CCT TCT TCG CCT TAA AGC	57 °C	[42]
			IGSb	AGC TCT TAT TCG CTG ATG GTA		
*Bartonella* spp.	*ssrA*	257	ssrA-F	GCT ATG GTA ATA AAT GGA CAA TGA AAT AA	60 °C	[43]
			ssrA-R	GCT TCT GTT GCC AGG TG		
*Rickettsia* spp.	*gltA* nested	381	RpCS.877p RpCS.1258n	GGG GAC CTG CTC ACG GCG G ATT GCA AAA AGT ACA GTG AAC C	58 °C	[44]
		338	RpCS.896p	GGC TAA TGA AGC AGT GAT AA		
			RpCS.1233n	GCG ACG GTA TAC CCA TAG C		
*Wolbachia* spp.	*wsp*	590–632	wsp81F	TGG TCC AAT AAG TGA TGA AGA AAC	55 °C	[8] modified
			wsp691R	AAA AAT TAA ACG CTA CTC CA		
Piroplasms						
*Babesia/Theileria* spp.	18S rRNA	433–489	BJ1	GTC TTG TAA TTG GAA TGA TGG	58 °C	[45]
			BN2	TAG TTT ATG GTT AGG ACT ACG		
Hymenopterans	28S rRNA	560	28s-hym-F	AGACCGATAGCGAACAAGTA	59 °C	[46]
			28s-hym-R	GGTCCTGAAAGTACCCAAA		
*Iph. hookeri*	*CO1*	268	401F	TTTAGAATATTTATTGATTCAGGGACT	53 °C	[8]
			44R	CTCCTGCTAAAACTGGTAAAGATAAT		

The selected positive PCR products, with amplified fragments of wasp- and/or pathogen-related genes, were purified using the NucleoSpin Gel and PCR Cleanup kit (Macherey-Nagel GmbH & Co., Duren, Germany) and Sanger sequenced in both directions using the corresponding PCR primers in the Laboratory of Biomedical Microbiology and Immunology (University of Veterinary Medicine and Pharmacy in Košice, Slovakia) and/or by Eurofins Genomics (Ebersberg, Germany).

Contiguous sequences were assembled and inspected for errors using MEGA 11 software [47]. The analyses of the assembled sequences were performed with BLASTn via GenBank. Nucleotide sequences were aligned using Clustal W and checked for the presence of polymorphisms within overlapped, analyzed gene fragments.

### 2.5. Statistical Analysis

Cross-tabulations were used to analyze the correlation between the presence of *Iph. hookeri* parasitoids and the presence of pathogens (*Wolbachia*, *Borreliella*, *Anaplasma*, *Rickettsia*, *Babesia*, and *Bartonella*) on *I. ricinus*, *H. concinna*, *H. inermis*, *D. reticulatus*, and *D. marginatus* ticks separately.

Pearson’s Chi-square, Maximum Likelihood Chi-square, Yates’ Correction of Chi-square, and Phi-square tests were used to test the measure of association. A *p*-value less than 0.001 determined a significant relation between variables (in the case of the first three Chi-square coefficients). The Phi-square value can range from 0 (no relation between factors) to 1 or −1 (perfect positive or negative relation between the two factors in the table). We reported Yates’ Correction of Chi-square (which is recommended for 2 × 2 tables) and Phi-square coefficients.

The analyses were conducted using StatSoft, Inc. (2013), STATISTICA (data analysis software system), version 12 (www.statsoft.com, accessed on 1 January 2021).

### 2.6. Calculation of Odds Ratio

The Odds ratio values were calculated using the calculators online available (https://select-statistics.co.uk, accessed on 1 January 2022) to determine the likelihood of pathogen accumulation in ticks that were infested by wasps (the co-occurrence of *Iph. hookeri* and tick-borne pathogens in various tick species).

## 3. Results

### 3.1. Molecular Identification of Iph. hookeri

To determine the presence and prevalence of endoparasitic wasps in an area with a sympatric occurrence of several epidemiologically important tick species, six out of seven species that are known to be present there were examined. The presence of endoparasitic wasps was recorded in questing juvenile *I. ricinus* and *H. concinna* ticks and in one *D. reticulatus* male from vegetation (Table 2 and Table 3). Moreover, partially engorged larvae of *D. marginatus* (n = 3) and *D. reticulatus* (n = 12) were removed from *Apodemus* spp. and *Clethrionomys* spp. trapped at the site and tested positive for *Iph. hookeri* (Table 4).

### 3.2. The Presence of Iph. hookeri and the Prevalence of Pathogens in Questing I. ricinus Ticks

Wasps were only found in *I. ricinus* nymphs, and all nymphs that were parasitized by a wasp carried DNA from *Wolbachia* spp. Tick-borne pathogens were detected in 46.43% (n = 280/603) of all examined *I. ricinus* ticks, with *Borreliella* spp. being the most prevalent, followed by *Bartonella* spp., *Rickettsia* spp., *Anaplasma* spp., and *Babesia* spp. Out of the 100 pools of examined larvae, 38 tested positive for tick-borne microorganisms. The most prevalent were *Rickettsia* spp., followed by an equal infection rate for *Anaplasma* spp. and *Bartonella* spp. Additionally, two pools of larvae tested positive for *Babesia* spp., but these could not be closely determined into species.

The spectrum of pathogens detected in the ticks infested by wasps was limited to the presence of three genera of bacteria, with the most prevalent being *Bartonella* spp., followed by *Borreliella* spp. and *Rickettsia* spp.

Ticks not infested by *Iph. hookeri* were found to carry a wider spectrum of pathogens compared to those parasitized by a wasp. The overall prevalence of pathogens in all *I. ricinus* ticks that were not infested by a wasp was highest for *Borreliella* spp., followed by *Bartonella* spp., *Rickettsia* spp., *Anaplasma* spp., and *Babesia* spp. (Table 2).

*Babesia microti* (PP086648 and PP086656) was identified in most of the females and nymphs of *I. ricinus*. *Rickettsia helvetica* (PP230811-PP230815) and *Rickettsia* sp. (PP230816) closely related to *R. monacensis* were detected in larvae, nymphs, and adult *I. ricinus* ticks. Regarding *Bartonella* spp., *I. ricinus* ticks carried DNA from *B. bovis* (PP230802-PP230805), as well as a *Bartonella* sp. closely related to *B. grahamii* (PP230809). All the samples were examined for the presence of *B. miamotoyi*, which was not confirmed in any of the tested samples. Several randomly chosen *Borreliella* sp.-positive samples were sequenced, predominantly representing *B. garinii* and *B. afzelii* (PP230799). All the samples that tested positive for *Borreliella* spp. in this study are referred to here as Lyme disease-causing borreliae of the sensu lato complex.

### 3.3. The Presence of Iph. Hookeri and the Prevalence of Pathogens in Questing H. concinna Ticks

*Haemaphysalis concinna* nymphs represented the stage of tick most infested by wasps, and all infested nymphs carried DNA from *Wolbachia*. Interestingly, two *H. concinna* females and a single nymph that tested negative for the presence of wasps were found to carry DNA from *Wolbachia* spp. In addition, two pools of larvae tested positive for *Iph*. *Hoookeri*.

Tick-borne pathogens were found in 13.67% (n = 41/300) of the examined *H. concinna* ticks, with *Babesia* spp. being the most prevalent pathogen, followed by *Bartonella* spp., *Rickettsia* spp., and *Anaplasma* spp. No *Borreliella* spp. were detected in any of the tested *H. concinna* ticks. Out of 94 pools of larvae tested, 6 carried DNA from tick-borne microorganisms, namely *Rickettsia* spp. and *Babesia* spp.

Only one *H. concinna* nymph that was parasitized by a wasp tested positive for *Anaplasma* spp., and one pool of larvae tested positive for *Rickettsia* spp.

*Haemaphyslis concinna* nymphs that were not infested by wasps carried the DNA of *Babesia* spp., *Bartonella* spp., and *Rickettsia* spp. Similarly, pools of larvae that were not infested by wasps carried DNA from *Rickettsia* spp. and *Babesia* spp. (Table 2).

The sequencing of positive amplicons confirmed the presence of *R. helvetica* (PP230818 and PP230819) and a *Rickettsia* sp. (P230817) closely related to the endosymbiont *Cand*. *R. mendelei*, *Babesia canis* (PP086528 and PP086530), a *Babesia* sp. closely related to *Babesia* sp. 2 (Eurasia) (PP086565), *Theileria capreoli* (PP086524, PP086526, PP086532, PP086645, PP086646, PP086675, and PP086676), and a *Babesia* sp. (PP213167) closely related to *Babesia motasi*.

### 3.4. The Presence of Iph. hookeri and the Prevalence of Pathogens in Questing and Engorged H. inermis Ticks

All the *H. inermis* ticks examined for the presence of parasitoids carried DNA from *Iph. hookeri*. At the same time, all the ticks tested negative for *Wolbachia* spp. However, the presence of *Rickettsia* spp., *Anaplasma* spp., and *Bartonella* spp. was recorded in this tick species.

Additionally, two larvae were removed from *Apodemus* and *Clethrionomys* spp. Although it tested negative for *Iph. hookeri*, one larva carried DNA from a *Babesia* sp. (Table 3) closely related to a strain previously isolated from *H. concinna* ticks, e.g., the Irkutsk region in Russia (KT725852).

### 3.5. The Presence of Iph. hookeri and the Prevalence of Pathogens in Questing and Engorged D. marginatus Ticks

Molecular screening for the presence of *Iph. hookeri* and *Wolbachia* spp. in this tick species was conducted, with negative results. These ticks were infected to a significant extent with SFG *Rickettsia* spp. Moreover, they carried DNA from *Anaplasma* spp., followed by *Borreliella* spp. and *Bartonella* spp., mainly represented by *B. grahamii* (PP230800 and PP230806-PP230808).

In addition to this, a total of 95 larvae were collected from wild rodents. Out of these, three larvae from small mammals tested positive for *Iph. hookeri* and *Wolbachia* spp. Additionally, one of these three larvae tested positive for *Babesia* spp. Among the engorged larvae, the highest prevalence was observed for *Borreliella* spp. and *Rickettsia* spp., followed by *Bartonella* spp. (Table 3).

### 3.6. The Presence of Iph. hookeri and the Prevalence of Pathogens in Questing and Engorged D. reticulatus Ticks

Among the 57 *D. reticulatus* ticks collected from vegetation, only one male tested positive for the presence of wasp DNA and *Rickettsia* spp. In wasp-free ticks from vegetation, the SFG *Rickettsia* spp. was the most prevalent microorganism followed by *Anaplasma* spp. Multiplex rt-PCR confirmed *B. microti* in a single specimen, while a *Borreliella* sp. and *Bartonella* spp. were not detected in any of the DNA samples.

In total, 16.67% of *D. reticulatus* larvae removed from wild rodents carried DNA from *Iph. hookeri*, and all but one tested positive for *Wolbachia* spp. The presence of *Babesia* spp., *Borreliella* spp., *Rickettsia* spp., and *Bartonella* spp. was confirmed in these engorged larvae.

The spectrum of pathogens detected in larvae from rodents infested by a wasp was narrower compared to ticks without wasps.

Ticks from rodents that were not affected by the presence of a wasp carried DNA from *Rickettsia* spp., *Babesia* spp., *Borreliella* spp., and *Bartonella* spp. (Table 4). The most prevalent species observed in the positive samples was closely related to *B. grahamii* (PP230810).

### 3.7. The Presence of Iph. hookeri and the Prevalence of Pathogens in Engorged I. trianguliceps Ticks

In total, 19 unfed or partially engorged *I. trianguliceps* larvae were removed from the small mammals of the genus *Apodemus*. All of them tested negative for *Iph. hookeri*, and all other microbes, except one partially engorged larva, tested positive for SFG *Rickettsia* spp.

### 3.8. Genetic Variation, Phylogenetic Analyses, and Nucleotide Sequences Obtained in the Study

All *cox1* nucleotide sequences of *Iph. hookeri* with a length of ± 270 bp obtained from different tick species in this study were identical with each other and showed 100% (97% query coverage/QC) identity with an *I. hookeri* Gardouch isolate from *I. ricinus* collected in France (JQ315225), and 87.55% (98% QC) similarity with the Encyrtidae sp. isolate BIOUG04746-H09 (KY830571) from Pakistan. In *cox1*-positive samples, an additional genetic marker, a portion of ribosomal 28S rRNA, was analyzed [48]. Partial 28S rRNA nucleotide sequences of the hymenopterans obtained from *I. ricinus* and *H. concinna* nymphs from the study were identical to each other in the overlapping region. On the other hand, several polymorphisms were detected in the partial hymenopteran 28S rRNA sequence from the *D. marginatus* larva removed from *Apodemus agrarius* after its comparison with sequences from wasps from *I. ricinus* and *H. concinna* nymphs.

The similarity between *Iph. hookeri* 28S rRNA fragments from *D. marginatus* and homologous sequences from *H. concinna* and *I. ricinus* was 98.2% and 98.5%, respectively.

It was confirmed that the 28S rRNA nucleotide sequences obtained from *I. ricinus* and *H. concinna* in this study are identical to the isolate from *I. ricinus* (OQ316577) from northwestern Hungary, close to the borders of Austria and Slovakia. They are also identical to the GP15 genotype found in *Rhipicephalus microplus*, *Ixodes persulcatus*, *Dermacentor silvarum*, and *H. concinna* (MN956813) ticks collected in Western Africa and near the city of Khabarovsk in Far Eastern Russia. Furthermore, the analysis revealed that there is a 99.42% similarity with *I. hookeri* Ixo4 (MH077537) previously isolated in Slovakia from *I. ricinus* [48]. Nearly all ticks infested with wasps carried the DNA of *Wolbachia pipientis*.

The nucleotide sequences obtained in this study were deposited in the GenBank database under the following accession numbers: for *Iph. hookeri cox1* (PP079105-PP079111); for *Iph. hookeri* 28S rRNA (PP084992-PP085022) (Table 5).

### 3.9. Molecular Detection of Microbes in Ticks

**Figure 1:**
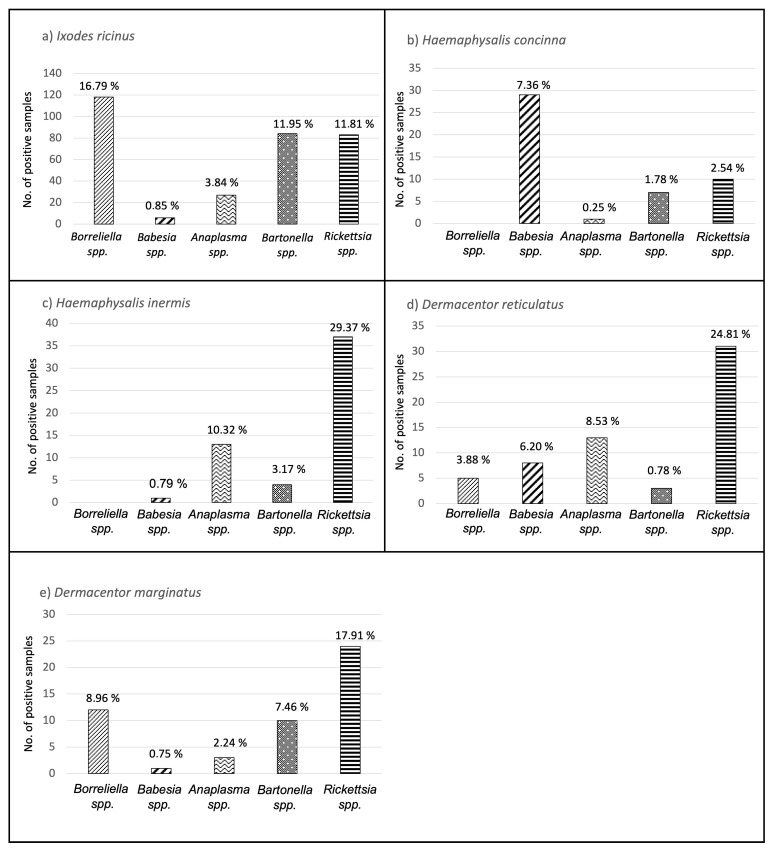
Spectrum of tick-borne pathogens in different tick species. (**a**) *I. ricinus*; (**b**) *H. concinna*; (**c**) *H. inermis*; (**d**) *D. reticulatus*; (**e**) *D. marginatus.*

The column graphs in Figure 1. indicate that *I. ricinus*, *D. reticulatus*, and *D. marginatus* ticks carried a wider spectrum of pathogens, with *Borreliella*, *Rickettsia*, and *Bartonella* (in *I. ricinus*) being the most detected. *Babesia*/*Theileria* spp. were predominantly found in *H. concinna* ticks, while *Rickettsia* spp. were most often detected in *H. inermis* ticks. The *Ixodes trianguliceps* ticks included in this study tested negative for all microbes, except for one specimen, which tested positive for *Rickettsia* spp.

### 3.10. Statistical Evaluation of the Correlations between the Presence/Absence of Wasps and Microbes in Screened Ticks

A significant association was confirmed between *I. hookeri* and *Wolbachia* spp. for all tick species included in this study (*I. ricinus*: Yates’ Correction of Chi-square = 637.5, df = 1, *p* ≤ 0.0001; Phi = 0.98; *H. concinna*: Yates’ Correction of Chi-square = 266.7, df = 1, *p* ≤ 0.0001; Phi = 0.57; *D. reticulatus*: Yates’ Correction of Chi-square = 96.7, df = 1, *p* ≤ 0.0001; Phi = 0.91; *D. marginatus*: Yates’ Correction of Chi-square = 85.9, df = 1, *p* ≤ 0.0001; Phi = 1.0).

This study found a significant correlation between *I. hookeri* and *Bartonella* spp. in *I. ricinus* (Yates’ Correction of Chi-square = 7.43, df = 1, *p* ≤ 0.0001; Phi = 0.12). In *H. concinna*, a correlation between *I. hookeri* and *Anaplasma* spp. was observed (Yates’ Correction of Chi-square = 3.66, df = 1, *p* ≤ 0.0001; Phi = 0.21). Similarly, a significant association between *I. hookeri* and *Babesia* spp. was confirmed in *D. marginatus* (Yates’ Correction of Chi-square = 9.75, df = 1, *p* ≤ 0.0001; Phi = 0.57)

### 3.11. Calculation of Odds Ratio

The findings show that in *I. ricinus* ticks, the presence of the *Iph. hookeri* wasp increased the probability of tick-borne pathogen accumulation by 2.11 times compared to ticks without the wasp. In *D. reticulatus*, the odds ratio of 1.46 indicated a higher likelihood of co-occurrence of wasps and pathogens. On the other hand, for *D. marginatus* and *H. concinna*, odds ratios of 0.92 and 0.66 suggested a decreased likelihood of co-occurrence, respectively (Table 6).

Six different species were tested for the presence of *Iph. hookeri*, *Wolbachia* spp., *Borreliella* spp., *Anaplasma* spp., *Bartonella* spp., and *Rickettsia* spp. In four species, the presence of *Iph. hookeri* was confirmed. Odds ratios >1 and <1 indicate the increased and decreased co-occurrences of wasps and pathogens, respectively.

## 4. Discussion

Studies have shown that *Ixodiphagus* spp. can potentially reduce tick populations, and as such, have been considered a promising candidate for tick control since their discovery in 1907 by Howard [17,49,50]. Most attempts to control ticks through parasitoid wasps were carried out about 100 years ago, with limited information about the biology of the wasps and their interactions with ticks [22,51,52]. Therefore, these attempts were not successful, most likely because the strains of parasitoid wasps from different geographic regions were not adapted to the local tick species, the seasonal pattern of occurrence, and the climatic conditions in which they were introduced (e.g., *Iph. hookeri* strains from Texas, USA released in South Africa, Italy, and Portugal) [53]. Among the more successful attempts to reduce tick populations using parasitoid wasps was the release of the local *Iph. hookeri* wasps on Naushon Island (USA) and in Kenya [54,55].

In Europe, *Iph. hookeri* has been confirmed in the Czech Republic [56], Ukraine [48], France [8,57], Georgia [58], Germany [19,59,60], the Netherlands [6,61], Finland [62], Italy [51], the United Kingdom [63], and Hungary [18]. In 1992, the parasitoid wasp *Iph. hookeri* was identified in three locations in Slovakia. It was found in the western part of the country and in the Slovak Karst region, in *H. concinna* and *I. ricinus* [27,31,64,65]. Our study brings the first molecular identification of *Ixodiphagus* wasps in *I. ricinus* and *H. concinna* ticks from Karst region and the first molecular detection of parasitoids in two *Dermacentor* species. The occurrence of various tick species with a nearly year-round activity and the wide range of hosts for tick development in this area creates a favorable environment for the wasps. Previously, ticks were collected here by Buczek et al. [31]. The authors reported a high percentage of *I. ricinus* (27.78%) and *H. concinna* (10.64%) nymphs infected with endoparasitic wasps. In our study, a lower prevalence of 7.0% and 7.74% for *Iph. hookeri* in *I. ricinus* and *H. concinna* were observed, respectively.

In western Slovakia, 13.8% of 50 examined *I. ricinus* nymphs were found to be infested by wasps [27]. In Lower Saxony, Germany, 1.86% to 3.8% of *I. ricinus* nymphs carried wasps, while in the Baden-Württemberg region, the rate of infestation was 2.41% to 3.24% [19]. In the southwest of the Netherlands, wasps were detected in 4% to 26% of *I. ricinus* nymphs, and in the northwest, the rate was 0.1% to 16% [37,61]. In the western part of France, 3.2% to 12.5% of *I. ricinus* ticks removed from roe deer and 19.6% to 20% of ticks from deer and vegetation in the southwest of the country tested positive for *Iph. hookeri* [8]. In Italy, 8.2% of *I. ricinus* ticks were found to be infected with a wasp [51], while in Finland, the rate of infestation was 0.4% to 2.3% in *I. ricinus* nymphs [62].

In Hungary, Tóth et al. [18] identified the southernmost detection point of *Iph. hookeri* in Central Europe. The authors found wasps in *I. ricinus* nymphs from 5 out of 21 screened sampling sites across the country. The authors compared 28S rRNA isolates from several sites in Hungary with an isolate (MH077537) obtained from *Iph. hookeri*-positive *I. ricinus* ticks from western Slovakia [46]. In our study, we found that the 28S rRNA sequences from *I. ricinus* and *H. concinna* from the Slovak Karst, which is close to several sampling sites in northern Hungary, where *Iph. hookeri*-infested ticks were not confirmed by Tóth et al. [18], are 100% identical with the isolate “Ihc” (OQ316577) from the western part of Hungary. This site borders Slovakia, where the *Iph. hookeri* 28S rRNA isolate Ixo4 (MH077537) was obtained by Gaye et al. [46]. In *H. concinna* and *I. ricinus* isolates from the Slovak Karst area, we identified at least three polymorphic sites previously described in Hungarian isolates, suggesting their genetic similarity.

The presence of *Iph. hookeri* was found to be positively correlated with the density of questing ticks and hosts, especially from the family Cervidae [37,54]. However, no correlation was found between the prevalence of *Iph. hookeri* in ticks and small rodents, which are important hosts for juvenile ticks.

In our study, 2.4% and 16.7% of *D. marginatus* and *D. reticulatus* larvae removed from small mammals (*Apodemus* spp. and *Clethrionomys* spp.) and one *D. reticulatus* male collected from vegetation tested positive for *I. hookeri*, respectively. *Dermacentor* spp. larvae and nymphs are nidicolous, feeding on the small mammals in their nests or burrow corridors. The association between the wasps’ infestation of nidicolous *Dermacentor* ticks and infection with rodent-associated pathogens suggests that wasps of this population are looking for the tick hosts while feeding on the rodents. The spectrum of rodent-related pathogens detected in juvenile *Dermacentor* ticks confirms this life strategy (Table 3 and Table 4).

A similar association was observed in the case of wasp-infested *I. ricinus* nymphs. These tested positive for *Bartonella* spp. (*B. grahamii*), *Borreliella* spp. (mostly *B. garinii* and *B. afzelii*), and *Rickettsia* spp. (mostly *R. helvetica*), suggesting that nymphs were injected by the wasps most probably while feeding on rodents. However, as we were not able to determine each pathogen at the species level, we cannot rule out the possibility that wasps injected ticks while feeding on other vertebrate hosts, such as deer. Several *I. ricinus* nymphs carried the DNA of *B. bovis*, which clusters phylogenetically with other ruminant-associated *Bartonella* species [66,67].

This is the first record of *Iph. hookeri* in two *Dermacentor* species in Slovakia and Central Europe. Previous studies from Europe suggested that this wasp preferred *I. ricinus* and *H. concinna* ticks, but the tick host preference can vary among *Iph. hookeri* populations [19,51]. For instance, the *Iph. hookeri* population in Germany did not lay eggs in *D. reticulatus* ticks, even in a no-choice situation under experimental conditions, unlike wasps from the former USSR [68]. In our study, the infested *Dermacentor* spp. ticks were removed as partially engorged larvae from small mammals, except for one questing male. The 28S rRNA genotype from *D. marginatus* was like the GP15 (MN956813) genotype from different tick species, including *H. concinna* and *D. silvarum*, from the Khekhtsir forest in Russia [46].

*Haemaphysalis concinna* ticks from our study carried several *Babesia* spp., which were highly similar to *Babesia* sp.2 (Eurasia) and some other isolates from Far Eastern Russia, (e.g., KH-Hc222/KT725849 or KJ486568). Additionally, previously obtained *B. canis* sequences from *I. ricinus* from western Slovakia clustered together in a well-supported clade with *B. canis* (AY649326), which was identified from *D. reticulatus* collected in Russia. Based on these similarities, we assume the phylogenetic relatedness and similarity of *Iph. hookeri* populations found in ticks from Slovakia with isolates from Russia, where the presence of *Iph. hookeri* has been confirmed in *H. concinna*, *D. reticulatus,* and *D. silvarum* ticks. Moreover, *D. silvarum* and *D. marginatus*, in which the presence of *Iph. hookeri* was also confirmed in our study, are considered closely related species, sharing a lot of certain characteristics [69].

As observed by Collatz et al. [19], *Iph. hookeri* populations exhibit significant variations based on their region, indicating that they are well adapted to local conditions. Our study found that different strains of *Iph. hookeri* can circulate among various tick species in the same area, but further research is required to confirm this hypothesis. Additionally, the presence of *Wolbachia* spp. in ticks infested by wasps suggests that it may be related to the presence of *Iph. hookeri* in ticks [61]. This theory has already been proposed in previous studies [5,8].

The study by Bobo [70] showed that the presence of *Wolbachia* significantly altered the native microbiome of ticks. Although ticks themselves do harbor the *Wolbachia* endosymbiont, it is present at a very low number. In our study, only three *H. concinna* ticks that were not infested by wasps carried the DNA of *Wolbachia* spp. Bobo [70] suggested that this may be due to a relationship between *Wolbachia* and Rickettsial endosymbionts in ticks. *Ixodes* ticks are often colonized by members of the genus *Rickettsia*, which is closely related to *Wolbachia* [71]. Bobo et al. [70] reported that the presence of two reproductive manipulators, *Wolbachia* and *Rickettsia*, places a heavy burden on the host. Thus, *Wolbachia* and *Rickettsia* exclude each other, which is why *Ixodes* ticks are negative for *Wolbachia* spp. but positive for *Rickettsia* spp. We observed a similar phenomenon in our study. Moreover, it is hypothesized that *Wolbachia* in ticks almost eliminates the presence of other endogenous microbes, which corresponds with the results of our study in the case of *H. concinna* ticks. In ticks, *Wolbachia* can promote colonization by *Iph. hookeri.* This approach involves manipulating the host immune system to promote symbiont proliferation and egg maintenance [8]. The presence of *Wolbachia* could induce an immune reaction in the tick that could eliminate, or harm, other microorganisms hosted by the tick, or on the contrary, it could create a suitable environment for some other microbes. However, more factors, including other members of microbiota as well as the various relationships among them, might be “behind the scenes”.

During our research, six tick species with different abilities and capacities to carry different groups and species of pathogens were examined. In *I. ricinus* and *Dermacentor* spp. ticks, it was observed that the presence of *Wolbachia* eliminated the presence of *Rickettsia* but did not influence the presence of other pathogens. The calculated values of the odds ratio also supported these results. However, the relationships and associations among pathogens in other tick species need to be further investigated.

In addition to maternally inherited bacteria, several other members of the microbiome have been observed to interact with tick-borne pathogens by either facilitating or inhibiting pathogen colonization and transmission. The results obtained by Lejal et al. [5] suggested that most correlations between the microbes are related to the presence of tick-borne pathogens (TBPs), the response to the parasitoid, or the seasonal effect.

Our study’s most prevalent microorganisms detected in ticks belonged to the genus *Rickettsia* spp. This was confirmed in each of the tested tick species, with the highest prevalence in *I. ricinus*, followed by *H. inermis*, *D. reticulatus*, *D. marginatus*, *H. concinna*, and *I. trianguliceps*. The most common species detected in *I. ricinus* and *H. concinna* ticks was *R. helvetica*, and members of the spotted fever group (SFG) rickettsiae in *D. marginatus*, *D. reticulatus*, and *H. inermis* ticks.

Ouarti et al. [72] confirmed the presence of *R. raoulti* in *D. reticulatus* and *H. inermis* ticks from the Karst area. Additionally, Heglasová et al. [32] screened tissue samples from 250 rodents of several species from the Slovak Karst and found a high prevalence of *Rickettsia* spp., some of which are considered pathogenic to humans. *Rickettsia raoulti* and *R. slovaca* are responsible for tick-borne lymphadenopathy/*Dermacentor*-borne necrosis erythema, lymphadenopathy/scalp eschar, and neck lymphadenopathy [71,72,73,74,75]. Diseases caused by rickettsiae are quite commonly reported in Slovakia [67,76,77]. As mentioned above, in this study, we confirmed the presence of *R. helvetica* in a great proportion of the examined *I. ricinus* ticks. Similarly, a high proportion of *D. reticulatus* and *H. inermis* ticks in our study carried the DNA of SFG *Rickettsia* spp., most presumably the species with zoonotic potential, such as *R. raoulti* and/or *R. slovaca*.

*Borreliella burgdorferi* sensu lato spirochetes have previously been found in questing and feeding *I. ricinus* (*B. garinii* and *B. afzelii*), *D. reticulatus* (*B. afzelii*), and *H. inermis* (*B. valaisiana*) ticks, with a significant predominance of genotypes associated with rodents and birds. Heglasová et al. [33] recently confirmed the presence of *Borrelia miyamotoi*, a relapsing fever group member, in *I. ricinus*, *D. marginatus*, *D. reticulatus*, *H. concinna*, and *H. inermis* from the Karst area. *Borreliella* spp. were found in over 20% of all *I. ricinus* nymphs tested in our study. Wasps infested several *I. ricinus* nymphs carrying spirochete DNA, suggesting that these parasitoids may interfere with the spread of *Borreliella* genotypes that cause Lyme disease [78,79]. Moreover, *D. marginatus* and *D. reticulatus* ticks from the sampling site have been shown to harbor *Borreliella* spp. as well. The majority of *Dermacentor* spp. ticks carrying spirochete DNA were found to be engorged larvae that were removed from small mammals; in many of these larvae, the presence of wasps was confirmed using PCR.

*Bartonella* spp. were the third most common microbe found molecularly in all tested tick species. *Bartonella bovis* was found in the majority of *I. ricinus* nymphs. This is the first report of the bacteria, which may cause endocarditis in cattle; the species has not yet been proven in Slovakia [80,81]. In this study, the presence of *B. grahamii*, which can cause cat scratch disease [82], and a *Bartonella* sp., which is closely related to *B. grahamii*, in *D. marginatus* and *D. reticulatus* ticks, was proved, respectively. *Bartonella grahamii* belongs to the most widespread species, occurring in wild mice (*Apodemus* spp.) and arvicolid voles (*Myodes* spp. and *Microtus* spp.) throughout Eurasia, from the UK to Japan [67,77,83,84,85], and it has been detected in several species of small mammals and ectoparasites in Slovakia as well [78,79].

*Anaplasma* spp. were found mostly in adult *I. ricinus*, *H. inermis*, *D. reticulatus*, and *D. marginatus* ticks and one *H. concinna* nymph. Blaňarová et al. [86,87] detected *A. phagocytophilum* in questing *I. ricinus* ticks and in rodent-feeding *I. trianguliceps* ticks, as well as in rodent biopsies, whereas it was not detected in rodents from the sites where *I. trianguliceps* ticks were absent. Phylogenetic analyses confirmed that *A. phagocytophilum* strains display specific host and vector associations and suggested that genotypes associated with rodents are probably transmitted solely by *I. trianguliceps* ticks, thus implying that rodent-associated strains may not pose a risk for humans. The blood meal of adult *I. ricinus* usually takes place on wild game animals, which are abundant in the study area. The *Anaplasma phagocytophilum* strains identified in questing *I. ricinus* ticks in this study showed a high similarity with several *msp2* isolates from questing *I. ricinus* ticks, deer, or dogs in the Genbank (e.g., MK802162, MK802159, MK625091, and MK570959). Ouarti et al. [72] found that 1.15% of *H. inermis* ticks from the Karst area carried bacteria from the family Anaplasmataceae. Interestingly, the prevalence of *Anaplasma* spp. in *H. inermis* ticks in our study reached more than 10%, suggesting its non-negligible role in the circulation of this bacteria.

Multiplex qPCR testing of *Dermacentor* spp. and *H. inermis* ticks showed that *Anaplasma* spp. in these tick species do not correspond to the GroEl1 or GroEl2 ecotypes.

We only found adult *Dermacentor* ticks from vegetation that contained *Anaplasma* spp. All the juvenile ticks from rodents tested negative, indicating that they do not carry the rodent-related *Anaplasma* ecotype. Our findings do, however, suggest that they are involved in the spread of *Anaplasma* spp. at the study location. It is worthwhile to genotype the bacteria in these tick species to better understand the roles of *Dermacentor* spp. and *H. inermis* in the spread of *Anaplasma* sp. ecotypes.

After screening several tick species from a site in the Karst region, Bona et al. [88] found that *Babesia* spp., which were only present in *I. ricinus*, were the second most common pathogen in the ticks they studied. Ticks carried mostly *B. microti*, the “Jena” strain, and *B. venatorum*. *Babesia* spp. were found in all examined tick species in our study, but primarily in *H. concinna* nymphs. *Theileria capreoli* and *Babesia* sp. 2 (Eurasia) were highly prevalent in *H. concinna* nymphs after sequencing the positive samples. These isolates are similar to those from questing and rodent-attached *H. concinna* ticks from western Slovakia (KU550688 and KU550689) or to the Kh-Hc222 isolate from *H. concinna* ticks from Russia (KJ486568). *Babesia motasi,* which is responsible for babesiosis in small ruminants, was detected in *H. concinna* larvae. In *I. ricinus* ticks, mostly rodent-associated *B. microti* was detected, and sporadically, *B. canis* was confirmed in questing nymphs.

The risk of numerous bacterial and/or protozoal diseases in this area is high, both for humans and animals. For wasps to develop, the tick nymph must begin to feed on a vertebrate host, which increases the risk of pathogen transmission. However, reducing the tick population can be achieved by using *Iph. hookeri* wasps, which kill infested nymphs after the emergence of adult wasps. This prevents the nymphs from molting into adult tick females or males and transmitting pathogens to vertebrate hosts. Tick females are also important vectors of different pathogens, including *Rickettsia* spp. and *Borreliella* spp. These bacteria are transmitted to thousands of larvae through transovarial transmission, with a transmission rate of up to 100% for some *Rickettsia* spp. [80,81] and more than 90% for *Borrelia miyamotoi* [82,89]. Consequently, when the wasps kill infected nymphs, the trans-stadially transmitted pathogens will not be transmitted to adult ticks or further spread to vertebrate hosts via adult ticks. Moreover, for some *Rickettsia* species as well as some relapsing fever borreliae, sexual transmission from a male to a female tick during copulation has been described [90,91]. *Ixodiphagus hookeri* can infest many tick species and has the potential to control tick populations and prevent the spread of parasites and pathogens in the Karst area.

## 5. Conclusions

Global climate change, together with various socio-demographic changes, creates urgent challenges related to vectors carrying pathogens and the need to develop control methods and measures. The fight against vectors, mosquitoes, and ticks often consists of the conventional use of acaricides, and alternative approaches based on biological control remain in the background. One of the methods of combating vectors is the use of parasitoid wasps of the genus *Ixodiphagus*. Monitoring the occurrence and host preference of wasps in areas with a sympatric occurrence of several epidemiologically important species of ticks that transmit specific types of pathogens and parasites is of indisputable importance from the point of protecting human and animal health. In this study, we identified the presence of wasps in four epidemiologically significant tick species of three genera. Many of them carry pathogens with the ability to cause serious diseases in humans and animals. Identifying and understanding tick-borne microbe interactions is essential for understanding the dynamics of vector-borne diseases and a precondition for the development of new strategies to control ticks and tick-borne diseases using the tick microbiome.

## Figures and Tables

**Table 2:** Prevalence of pathogens detected in questing *I. ricinus* and *H. concinna* ticks.

Prevalence of Pathogens Detected in Questing *I. ricinus* (%)										
Developmental Stage	Total No. of Examined Ticks	Infestation with *Iph. hookeri*	Prevalence (%) MIR * (%)	*Iph. hookeri*	*Wolbachia* spp.	*Borreliella* spp.	*Babesia* spp./*Theileria* spp.	*Anaplasma* spp.	*Bartonella* spp.	*Rickettsia* spp.
Male	154	Negative	Prevalence (%) (no. of positive ticks)	0	0	(n = 29) 18.83	0	(n = 14) 9.09	(n = 5) 3.25	(n = 14) 9.09
Female	149			0	0	(n = 34) 22.81	(n = 2) 1.34	(n = 8) 5.37	(n = 8) 5.37	(n = 16) 10.74
Nymph	279			0	(n = 1) 0.36	(n = 49) 17.56	(n = 2) 0.72	(n = 1) 0.36	(n = 60) 21.51	(n = 24) 8.60
	21	Positive		(n = 21) 100	(n = 21) 100	(n = 6) 28.57	0	0	(n = 7) 33.33	(n = 1) 4.76
Total no. of wasp-free ticks	582	Negative		0	(n = 1) 0.17	(n = 112) 19.24	(n = 4) 0.69	(n = 23) 3.95	(n = 73) 12.54	(n = 54) 9.28
Total	603		Overall prevalence (%)	(n = 21) 3.48	(n = 22) 3.65	(n = 118) 19.57	(n = 4) 0.66	(n = 23) 3.81	(n = 80) 13.27	(n = 55) 9.12
Larvae	100 pools of 10 larvae		MIR (%)	0	0	0	(n = 2) 2	(n = 4) 4	(n = 4) 4	(n = 28) 28
**Prevalence of Pathogens Detected in Questing *H. concinna* (%)**										
Female	3	Negative	Prevalence (%) (no. of positive ticks)	0	2/3	0	0	0	0	0
Nymph	274			0	(n = 1) 0.37	0	(n = 27) 9.85	0	(n = 7) 2.56	(n = 6) 2.19
	23	Positive		(n = 23) 100	(n = 21) 91.3	0	0	(n = 1) 4.35	0	0
Total no. of wasp-free ticks	277	Negative		0	(n = 3) 1.08	0	(n = 27) 9.75	0	(n = 7) 2.53	(n = 6) 2.17
Total	300		Overall prevalence (%)	(n = 23) 7.67	(n = 24) 8.00	0	(n = 27) 9.00	(n = 1) 0.33	(n = 7) 2.33	(n = 6) 2.00
Larvae	92 pools of 10 larvae	Negative	MIR (%)	0	0	0	(n = 2) 2.17	0	0	(n = 3) 3.26
	2 pools of 10 larvae	Positive		2/2	0	0	0	0	0	1/2
Total	94 pools		Overall MIR (%)	(n = 2) 2.13	0	0	(n = 2) 2.13	0	0	(n = 4) 4.26

* MIR—minimum infection rate.

**Table 3:** Prevalence of pathogens in questing *D. marginatus*, *D. reticulatus*, and *H. inermis* ticks.

Prevalence of Pathogens Detected in Questing *D. marginatus* (%)										
Developmental Stage	Total No. of Examined Ticks	Infestation with *Iph. hookeri*	Prevalence (%)	*Iph. hookeri*	*Wolbachia* spp.	*Borreliella* spp.	*Babesia* spp./*Theileria* spp.	*Anaplasma* spp.	*Bartonella* spp.	*Rickettsia* spp.
Male	12	Negative	Prevalence (%) (no. of positive ticks)	0	0	0	0	(n = 3) 25	(n = 1) 8.33	(n = 9) 75
Female	7			0	0	(n = 1) 14.29	0	0	0	(n = 4) 57.14
Nymph	8			0	0	(n = 1) 12.5	0	0	(n = 1) 12.5	(n = 1) 12.5
Larvae	12			0	0	0	0	0	0	0
Total	39		Overall prevalence (%)	0	0	(n = 2) 5.13	0	(n = 3) 7.69	(n = 2) 5.13	(n = 14) 35.9
**Prevalence of Pathogens Detected in Questing *D. reticulatus* (%)**										
Male	18	Negative		0	0	0	0	(n = 3) 15.79	0	(n = 10) 55.56
	1	Positive		1/1	0	0	0	0	0	1/1
Female	31	Negative	Prevalence (%) (no. of positive ticks)	0	0	0	(n = 1) 3.22	(n = 8) 25.81	0	(n = 14) 45.16
Nymph	7			0	0	0	0	0	0	(n = 2) 28.6
Total no. of wasp-free ticks	56	Negative		0	0	0	(n = 1) 1.79	(n = 11) 19.64	0	(n = 26) 46.43
Total	57		Overall prevalence (%)	(n = 1) 1.75	0	0	(n = 1) 1.75	(n = 11) 19.3	0	(n = 27) 47.37
**Prevalence of Pathogens Detected in Questing *H. inermis* (%)**										
Male	55	Negative	Prevalence (%) (no. of positive ticks)	0	0	0	0	(n = 7) 12.73	(n = 2) 3.64	(n = 25) 45.45
Female	60			0	0	0	0	(n = 5) 8.33	(n = 2) 3.33	(n = 3) 5
Nymph	9			0	0	0	0	(n = 1) 11.11	0	(n = 9) 100
Total	124		Overall prevalence (%)	0	0	0	0	(n = 13) 10.48	(n = 4) 3.23	(n = 37) 29.84

**Table 4:** Prevalence of pathogens in ticks from rodents.

Prevalence of Pathogens in Different Tick Species from Rodents (%)											
Tick Species	Developmental Stage	Total no. of Examined Ticks	Infestation with *Iph. hookeri*	Prevalence (%)	*Iph. hookeri*	*Wolbachia* spp.	*Borreliella* spp.	*Babesia* spp./*Theileria* spp.	*Anaplasma* spp.	*Bartonella* spp.	*Rickettsia* spp.
*I. trianguliceps*	Larvae	19	Negative		0	0	0	0	0	0	(n = 1) 5.26
*H. inermis*	Larvae	2	Negative		0	0	0	1/2	0	0	0
*D. marginarus*	Larvae	92	Negative	Prevalence (%) (no. of positive ticks)	0	0	(n = 10) 10.87	0	0	(n = 8) 8.71	(n = 10) 10.87
		3	Positive		3/3	3/3	0	1/3	0	0	0
		95	Total		(n = 3) 3.16	(n = 3) 3.16	(n = 10) 10.53	(n = 1) 1.1	0	(n = 8) 8.42	(n = 10) 10.53
*D. reticulatus*	Larvae	60	Negative		0	0	(n = 4) 6.67	(n = 6) 10	0	(n = 1) 1.67	(n = 2) 3.33
		12	Positive		(n = 12) 100	(n = 11) 91.67	(n = 1) 8.33	(n = 1) 8.33	0	0	(n = 3) 25
		72	Total		(n = 12) 16.67	(n = 11) 15.27	(n = 5) 6.94	(n = 7) 9.72	0	(n = 1) 1.39	(n = 5) 6.94

**Table 5:** Nucleotide sequences obtained in this study, which were submitted to the GenBank database.

Host	Organism	Gene Fragment	Accession Number
*I. ricinus*	*Iph. hookeri*	*cox1*	PP079108-PP079110
*I. ricinus*	*Iph. hookeri*	28S rRNA	PP085018-PP085022
*I. ricinus*	*B. microti*	18S rRNA	PP086648; PP086656
*I. ricinus*	*A. phagocytophilum*	*msp2*	PP239200-202
*I. ricinus*	*Borreliella afzelii*	rrfA-rrlB	PP230799
*I. ricinus*	*R. helvetica*	*gltA*	PP230811-PP230815
	*Rickettsia* sp.	*gltA*	PP230816
	*B. bovis*	*ssrA*	PP230802-PP230805
	*Bartonella sp.*	*ssrA*	PP230809
*H. concinna*	*Iph. hookeri*	*cox1*	PP079105-PP079107
*H. concinna*	*Iph. hookeri*	28S rRNA	PP084992-PP085001
*H. concinna*	*B. canis*	18S rRNA	PP086528; PP086530
*H. concinna*	*Babesia* sp. 2 (*Eurasia*)	18S rRNA	PP086565
*H. concinna*	*Th. capreoli*	18S rRNA	PP086524; PP086526; PP086532; PP086645-46; PP086675-76
*H. concinna*	*Babesia* sp.	18S rRNA	PP213167
*H. concinna*	*R. helvetica*	*gltA*	PP230818, PP230819
	*Rickettsia* sp.	*gltA*	PP230817
*D. marginatus*	*Iph. hookeri*	28S rRNA	PP085019
	*B. grahamii*	ssrA	PP230800, PP230806-PP230808
*D. reticulatus*	*Bartonella* sp.	*ssrA*	PP230810

**Table 6:** Observed co-occurrence of *Iph. hookeri* and tick-borne pathogens in ticks.

	*I. ricinus*	*H. concinna*	*D. marginatus*	*D. reticulatus*
Observed Co-occurrence	12	2	1	6
Odds ratio	2.12	0.66	0.92	1.46

## Data Availability

The original contributions presented in the study are included in the article, further inquiries can be directed to the corresponding author/s. The raw data supporting the conclusions of this article will be made available by the authors on request.
